# Rapid Tranquillisation Practice and Debriefing; an Observational Study in Adult Psychiatric Inpatients

**DOI:** 10.1192/bjo.2024.480

**Published:** 2024-08-01

**Authors:** Georgina James, Asif Mir

**Affiliations:** 1 Manchester Foundation Trust, Manchester, United Kingdom; 2 Greater Manchester Mental Health, Manchester, United Kingdom

## Abstract

**Aims:**

Rapid Tranquillisation (RT) is the administration of parenteral sedation to de-escalate situations where patients may harm themselves and/or others. RT is a restrictive intervention potentially breaching patients’ human rights and is reserved for situations where other measures have failed. NICE guidelines (NG10) state that once immediate risks are managed, post-RT incident debriefing of patients and staff should be conducted. This study examines concordance with NG10 at an adult inpatient psychiatric unit and explores ways to improve compliance and patient/staff experience.

**Methods:**

Adult psychiatric inpatients (aged 18–65) who received single or multiple RT therapy during the admission study period (October 2023) were included. Data collected from Electronic Patient Records and chart review included gender, age, ward type, date of RT and drug(s) administered. Following RT, anonymised data was collected on the presence, nature and details of debriefing.

**Results:**

49 adult psychiatry patients were admitted during the study period and there were 32 episodes of RT use in 9 patients (18.4% of inpatients). 56.7% of these occurred on general wards and 43.8% on psychiatry intensive care, with 6 patients (66.7%) >1 episode. Intramuscular drugs used included one or more of lorazepam (78.1% of patients), haloperidol (25%), promethazine (21.9%) and aripiprazole (9.4%). After 46.9% (15/32) of RT episodes debrief was offered, and occurred in 28.1% (9/32); 40% (6/15) of those offered debrief did not participate. 52.4% (11/21) of female patients were offered debrief with 81.8% (9/11) uptake, compared with 36.3% (4/11) of male patients offered with 25% (1/4) uptake. No accounts were taken from patients’ advocate, carer or witnesses. Details of debriefs conducted were documented in 33.3% (3/9). Reasons for not conducting debriefing were documented in 43.5% (10/23). The most common reason given was “not clinically appropriate”. During debriefs, no patients were offered information leaflets about the RT medication used. Finally, staff debriefing occurred in 31.3% (10/32) of episodes.

**Conclusion:**

Compliance with NG10 guidelines for debriefing inpatients following RT was low and was worse in male than female patients. Staff debriefing was poor and no witness or advocate accounts were utilised. These findings may be due to the stressful ward environment or disrupted patient-professional relationship immediately following RT administration. Understanding the reasons for the gender differences in uptake, patient, staff or environmental factors contributing to lack of debriefing will allow interventions and improve holistic patient care following RT. Information and awareness of RT therapy should be available for patients more readily on ward admission and following RT.

